# Impact of empagliflozin on cardiac structure and function assessed by echocardiography after myocardial infarction: a post-hoc sub-analysis of the emmy trial

**DOI:** 10.1007/s00392-024-02523-1

**Published:** 2024-09-16

**Authors:** Nora Schwegel, Christoph Strohhofer, Ewald Kolesnik, Sabrina Oltean, Alexander Hüttmair, Christian Pipp, Martin Benedikt, Nicolas Verheyen, Johannes Gollmer, Klemens Ablasser, Markus Wallner, Viktoria Santner, Norbert Tripolt, Peter Pferschy, Peter Zechner, Hannes Alber, Jolanta M. Siller-Matula, Kristen Kopp, Andreas Zirlik, Faisal Aziz, Harald Sourij, Dirk von Lewinski

**Affiliations:** 1 Division of Cardiology, University Heart Center Graz, Medical University of Graz, Graz, Austria; 2 Trials Unit for Interdisciplinary Metabolic Medicine, Division of Endocrinology and Diabetology, Department of Internal Medicine, Medical University of Graz, Graz, Austria; 3 Department of Cardiology and Intensive Care Medicine, Hospital Graz II, West Location, Graz, Austria; 4 Department of Cardiology, Public Hospital Klagenfurt Am Woerthersee, Klagenfurt Am Woerthersee, Austria; 5 Department of Cardiology, Medical University of Vienna, Vienna, Austria; 6 Division of Cardiology and Internal Intensive Care Medicine, Department of Internal Medicine II, Paracelsus Medical Private University of Salzburg, Salzburg, Austria

**Keywords:** Myocardial infarction, Echocardiography, SGLT2 inhibitors, Empagliflozin, Heart failure, Myocardial function

## Abstract

**Background:**

Empagliflozin administered after acute myocardial infarction proofed to improve cardiometabolic parameters and biomarkers, but the impact on cardiac function is still largely unknown. The aim of this post-hoc echocardiographic sub-analysis of the EMMY trial was to provide in-depth echocardiographic analysis on the effects of empagliflozin versus placebo on standard and novel echocardiographic structural and functional parameters after acute myocardial infarction.

**Methods:**

In this post-hoc analysis of the EMMY trial a subset of 313 patients (157 empagliflozin vs. 156 placebo) was enrolled for post-processing analysis of echocardiographic structural and functional parameters. On top of two-dimensional and Doppler parameters, myocardial deformation analyses were performed to assess ventricular and atrial strain values.

**Results:**

Left ventricular volumes showed significant differences in favor of empagliflozin over the course of the trial (change in left ventricular end-diastolic volume median [interquartile range] 8 [−3;19]% versus 13 [0;29]%, p = 0.048; left ventricular end-systolic volume −3 [−15;12]% versus 4 [−12;18]%, p = 0.044). This effect persisted after adjusting for baseline values, age, and sex. Left ventricular systolic and diastolic function overall improved over the course of the trial and parameters for diastolic function showed a distinct trend between groups but did not meet statistical significance in this cohort.

**Conclusion:**

In this post-hoc analysis among patients with acute myocardial infarction, treatment with empagliflozin resulted in a significant beneficial effect on left ventricular end-diastolic and end-systolic volume, without significantly improving left ventricular or right ventricular functional parameters compared to placebo after 26 weeks.

**ClinicalTrials.gov registration:**

NCT03087773.

**Graphical abstract:**

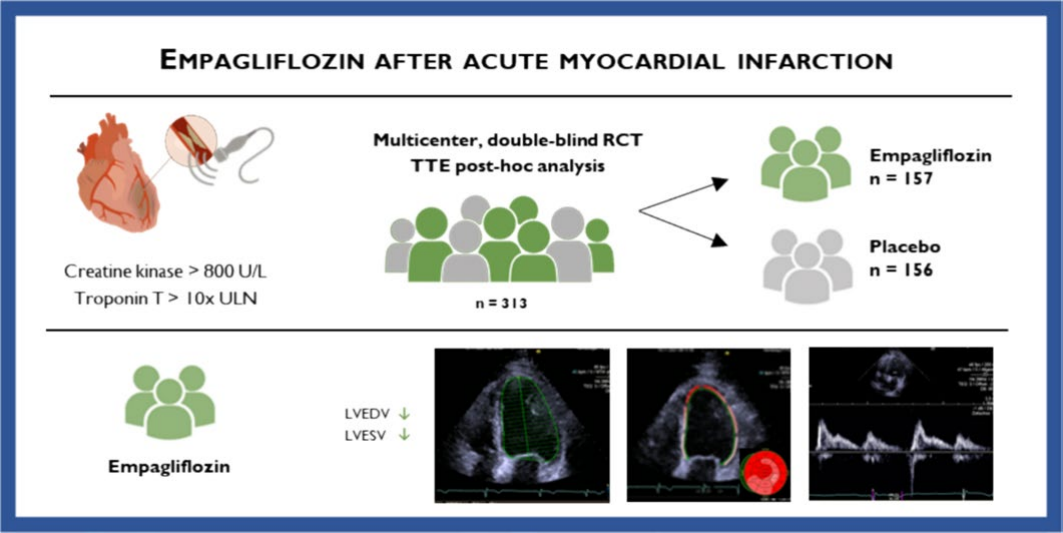

**Supplementary Information:**

The online version contains supplementary material available at 10.1007/s00392-024-02523-1.

## Introduction

Left ventricular remodeling and systolic dysfunction following acute myocardial infarction (AMI) increase the risk for the development of heart failure and overall mortality [1, 2]. Established heart failure therapies like angiotensin converting enzyme inhibitors (ACEi), angiotensin receptor blockers (ARB), angiotensin receptor neprilysin inhibitors (ARNI), and beta-blockers attenuate post-AMI ventricular remodeling and dysfunction and lead to a reduction in the risk of adverse events [1–4]. In the past years, sodium-glucose co-transporter 2 inhibitors (SGLT2i) gained ground as heart failure therapy and most recently their use was recommended for the treatment of heart failure over the whole spectrum of ejection fraction [5, 6]. The EMpagliflozin in acute MYocardial infarction (EMMY) trial investigated the effects of treatment with the SGLT2i empagliflozin versus placebo on N-terminal pro-hormone of brain natriuretic peptide (NT-proBNP) in patients after AMI. A total of 476 patients were randomized in a multi-center, double-blinded trial, with a follow-up period of 26 weeks. Treatment with empagliflozin was associated with a significantly greater reduction in NT-proBNP over 26 weeks (mean 26-week NT-proBNP 15% [95%CI −4.4 to −23.6%] lower in treatment group, p = 0.026). Moreover, an increase in left ventricular ejection fraction (LVEF) of 1.5% (95%-CI 0.2–2.9%, p = 0.029) difference between the groups in favor of empagliflozin was observed. Left ventricular end-systolic volume (LVESV) decreased with empagliflozin and increased in the placebo group, resulting in a −7.5 ml (95% CI −11.5 to −3.4, p = 0.0003) difference. Left ventricular end-diastolic volume (LVEDV) increased in both groups, with a difference of -9.7 ml (95% CI −15.7 to −3.7, p = 0.0015) in favor of empagliflozin. E/e’ decreased significantly more pronounced in the empagliflozin group (−6.8% [95% CI 1.3–11.3%], p = 0.015) [7]. However, these echocardiographic measurements were evaluated locally and comprehensive evaluations like deformation analysis were not performed. In the present post-hoc analysis of a subset of participants the EMMY trial, in-depth echocardiographic analysis was performed in an Echocardiographic Core Laboratory to meticulously assess the effect of empagliflozin on echocardiographic structural and functional parameters after large AMI.

## Methods

### The EMMY trial

EMMY was a multi-center, randomized, double-blind, placebo-controlled trial conducted at 11 Austrian sites (ClinicalTrials.gov registration nr. NCT03087773). From May 2017 to May 2022 a total of 476 patients with a confirmed AMI with creatine kinase > 800 IU/L, a high-sensitivity Troponin level > tenfold the upper limit of normal, and an estimated glomerular filtration rate (eGFR) > 45 mL/min/1.73 m^2^ were enrolled within 72 h after percutaneous coronary intervention (PCI) for AMI. Patients with an ongoing SGLT2i therapy or therapy within 4 weeks prior to enrolment were excluded. Empagliflozin (10 mg/day, target dose) versus matching placebo was administered on the background of guideline based post-MI therapy [8]. Follow-up visits were performed after 6, 12, and 26 weeks. Detailed information on trial design, baseline characteristics, and results of the trial have been previously published elsewhere [7, 9].

Comprehensive echocardiographic examination was performed according to current guidelines[10] on locally available devices at baseline, after 6 weeks, and after 26 weeks. The echocardiographic protocol for transthoracic echocardiography (TTE) included 2D, Doppler, and M-mode imaging. During each study an ECG was recorded to define end-systole and end-diastole. All examinations were analysed for standard parameters at local departments and the results, comprising left-ventricular volumes, LVEF, and E/e’, were reported in the main EMMY trial [7].

Additionally, all sites enrolling patients in the main trial were invited to transfer their echocardiographic examinations archived as DICOM-files to the Echocardiography Core Laboratory (Department of Cardiology, Medical University of Graz, Austria) to participate in the echocardiographic post-hoc sub-analysis. To qualify for participation, a predefined echocardiographic protocol, which was provided by the Echocardiography Core Laboratory, was followed by enrolling sites. An overview of the protocol is provided in the Online Resource. (Figure S1).

### The echocardiographic post-hoc sub-analysis

**Fig. 1:**
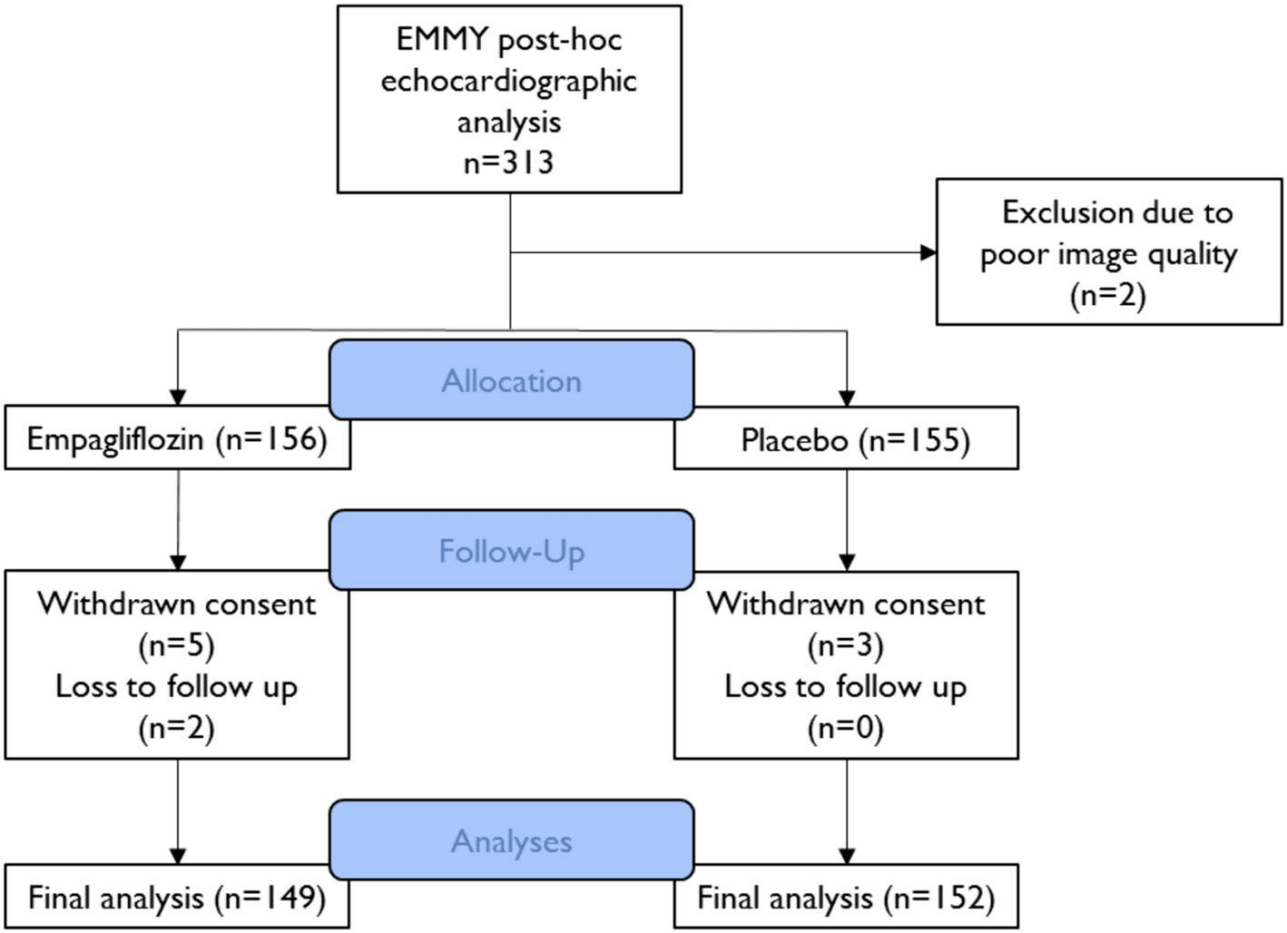
Patient disposition. Echocardiographic data for evaluation was available from three major sites: the Medical University of Graz (n = 219), the Hospital Graz II—West (n = 16), and the Hospital Klagenfurt am Woerthersee (n = 78)

In this analysis, we retrospectively assessed the archived studies of the echocardiographic examinations conducted during the EMMY trial from three major sites (see Fig. 1) in the central Echocardiography Core Laboratory. Analyses were performed using the vendor-independent post-processing software TomTec-Arena (TomTec Imaging Systems, Munich, Germany) by trained investigators who were blinded to patients’ clinical characteristics and study treatment. Two-dimensional and Doppler parameters were assessed according to current guidelines of the European Association of Cardiovascular Imaging and the American Society of Echocardiography [10–13].

### Deformation analysis

In addition, 2D deformation analyses were performed using 2D Cardiac Performance Analysis (2D-CPA) by TomTec-Arena. For this purpose, cine-loops with the best image quality were selected for each analysis, and deformation analysis was performed in two cardiac cycles and reported as mean values, if applicable. The integrity of tracking was confirmed by visual impression of wall motion, and contours were readjusted to enable optimal tracking.

In this study, we focused on endocardial strain parameters derived from user-defined contours. Strain values were calculated by using the entire contour line length. Left ventricular (LV) global longitudinal strain (GLS) was assessed from all three apical chamber views (LV four-chamber, three-chamber, and two-chamber view) using a 16-segment model [14, 15]. Right ventricular (RV) longitudinal strain was assessed by averaging longitudinal systolic strain values of septal and free wall segments (six segment model, RV global longitudinal strain; RV-GLS) as well as from the free wall segments alone (three segment model, RV freewall strain; RV-FWS), obtained from an RV focused apical four-chamber view [16]. Left atrial (LA) strain was obtained from apical two-chamber view, and right atrial (RA) strain was assessed in apical four-chamber view, in accordance with current recommendations [16].

All echocardiographic parameters and assessment methods are listed in detail in Online Resource Table S1*.*

### Statistical analysis

All data is illustrated by descriptive statistics using mean and standard deviation or median and interquartile range for continuous variables and frequency and percentages for categorical variables, respectively. Categorical variables were compared using the Chi-square test or Fisher’s Exact test and continuous variables were compared using unpaired t-tests or their non-parametric equivalent tests, as appropriate. The change in echocardiographic parameters from baseline to week 6 and week 26 as well as differences between treatment groups over the three timepoints were analyzed using the linear mixed-effects model. In each model, time, treatment, and time-treatment interaction were included as fixed effects along with baseline values of each parameter, age, and sex. P-values <0.05 were considered statistically significant.

## Results

A total of 313 patients initially qualified for this post-hoc analysis of the EMMY trial (empagliflozin n = 157 versus placebo n = 156). Eight (2.6%) patients withdrew consent, two (0.6%) patients were lost to follow up, and two (0.6%) patients were excluded due to poor image quality. Hence, 301 participants (empagliflozin n = 149, placebo n = 152) were included in the final analysis. (Fig. 1) Median (interquartile range) age was 57 (52;65) years, with 18.3% females. 37 (11.9%) patients had established diabetes mellitus, 17 (5.5%) patients had known coronary artery disease, and 12 (3.9%) had previous history of myocardial infarction. Baseline characteristics were similar between groups, as illustrated in Table 1. At baseline median NT-proBNP was 1377 (800;2217) pg/mL. Median creatine kinase was 1705 (1203;2442) U/L and median Troponin T was 3067 (2099;4938) µg/L. All patients received guideline recommended post-MI pharmacologic treatment with > 97% of patients receiving treatment with ACEi/ARB/ARNI, beta-blockers, and statins.

**Table 1:** Baseline characteristics

	All	Empagliflozin	Placebo	P-value^a^
	n = 311	n = 156	n = 155	
Age [years]	57 (52;65)	57 (52;64)	58 (52;66)	0.702
Female, *n (%)*	57 (18.3)	25 (16.0)	32 (20.6)	0.292
Body mass index [kg/m^2^]	28 (25;30)	28 (25;30)	28 (25;30)	0.591
Diabetes mellitus, *n (%)*	37 (11.9)	16 (10.3)	21 (13.5)	0.370
Coronary artery disease, *n (%)*	17 (5.5)	10 (6.4)	7 (4.5)	0.463
Coronary vessel status				
1-vessel disease, *n (%)*	143 (46.0)	62 (39.7)	81 (52.3)	0.063
2-vessel disease, *n (%)*	108 (34.7)	58 (37.2)	50 (32.3)	
3-vessel disease, *n (%)*	60 (19.3)	36 (23.1)	24 (15.5)	
History of myocardial infarction, *n (%)*	12 (3.9)	8 (5.1)	4 (2.6)	0.243
Pharmacologic treatment				
ACEi/ARB, *n (%)*	304 (98.7)	153 (98.7)	151 (98.7)	0.990
ARNI, *n (%)*	5 (1.6)	1 (0.6)	4 (2.6)	0.174
Beta-blocker, *n (%)*	303 (97.4)	150 (96.2)	153 (98.7)	0.155
MRA, *n (%)*	149 (47.9)	73 (46.8)	76 (49.0)	0.693
Loop diuretic, *n (%)*	33 (10.6)	18 (11.5)	15 (9.7)	0.594
Calcium channel blocker, *n (%)*	13 (4.2)	4 (2.6)	9 (5.8)	0.153
Statin, *n (%)*	310 (99.7)	155 (99.4)	155 (100.0)	0.318
Laboratory parameters				
NT-proBNP [pg/mL]	1377 (800;2217)	1257 (797;2239)	1477 (800;2192)	0.514
eGFR [mL/min/1.73 m^2^]	93 (79;102)	94 (78;101)	93 (81;103)	0.576
Creatine kinase [U/L]	1705 (1203;2442)	1670 (1170;2518)	1729 (1257;2366)	0.883
CK-MB [U/L]	159 (86;238)	139 (80.0;227)	167 (92;247)	0.373
Troponin T [µg/L]	3067 (2099;4938)	3089 (2195;4899)	3045 (2062;5018)	0.512
C-reactive Protein [mg/dL]	6 (3;14)	6 (3;13)	7 (2;14)	0.660
Echocardiographic parameters				
LV EDV [mL]	122 (100;142)	120 (100;140)	122 (101;145)	0.417
LV ESV [mL]	63 (49;78)	62 (49;77)	64 (49;80)	0.571
LV EF [%]	48 (43;53)	49 (43;52)	47 (43;53)	0.909
LV GLS [%]	-16 (-19;-13)	-17 (-19;-13)	-15 (-19;-12)	0.257
E/e’	9 (7;11)	9 (7;11)	9 (7;11)	0.559
LA GLS [%]	19 (15;25)	19 (15;25)	19 (14;26)	0.959
LAVI [mL/m^2^]	31 (27;38)	30 (26;37)	32 (28;39)	0.147
RV GLS [%]	-21 (-23;-18)	-21 (-23;-18)	-21 (-23;-18)	0.890
RV-FWS [%]	-27 (-30;-23)	-27 (-30;-23)	-27 (-29;-24)	0.886
RV FAC [%]	37 (34;41)	37 (35;42)	38 (33;41)	1.000
TAPSE [mm]	21 (19;23)	21 (18;23)	21 (19;23)	0.151
RA GLS [%]	35 (29;42)	35 (30;41)	35 (29;43)	0.640
RAVI [mL/m^2^]	23 (18;28)	22 (18;27)	23 (18;29)	0.752

All parameters reported in median (interquartile range) or frequency (percentage). *ACEi* angiotensin converting enzyme inhibitor, *ARB* angiotensin receptor blocker, *ARNI *angiotensin receptor neprilysin inhibitor, *CK-MB* creatine kinase muscle-brain type, *eGFR* estimated glomerular filtration rate, *LA-GLS* left-atrial global longitudinal strain, *LAVI* left-atrial volume index, *LVEDV* left-ventricular end-diastolic volume, *LVEF* left-ventricular ejection fraction, *LVESV* left-ventricular end-systolic volume, *LV-GLS* left-ventricular global longitudinal strain, *MRA* mineralcorticoid receptor antagonist, *RA-GLS* right-atrial global longitudinal strain; *RAVI* right- atrial volume index, *RV-FAC* right-ventricular fractional area change; *RV-FWS* right-ventricular freewall strain, *RV-GLS* right-ventricular global longitudinal strain, *TAPSE* tricuspid annular plane systolic excursion

^a^Wilcoxon rank-sum test; Chi-square test

Patients had mildly reduced LVEF with a median of 48 (43;53) % at baseline, preserved right ventricular function with a median tricuspid annular plane systolic excursion (TAPSE) of 21 (19;23) mm, and at median normal atrial volume indices (left atrial volume index [LAVI] 31 [27;38] mL, right atrial volume index [RAVI] 23 [18;28] mL).

### Changes in cardiac structure and function after 6 and 26 weeks

Changes in echocardiographic structural and functional parameters and corresponding treatment effects are shown in Table 2 and in the Online Resource *Table S3*.

**Table 2:** Changes in echocardiographic parameters

	Baseline	Week 6	Week 26	Absolute change (week 6)	Absolute change (week 26)	% change (week 6)	% change (week 26)	p−value
LVEDV [mL]								
All	122 (100;142)	132 (110;151)	135 (114;158)	6 (−4;21)	12 (−1;28)	5 (−3;18)	10 (−1;24)	
Empagliflozin	120 (100;140)	132 (113;149)	135 (114;155)	6 (−5;20)	10 (−3;20)	4 (−4;17)	8 (−3;19)	**0.048**
Placebo	122 (101;145)	132 (107;155)	134 (111;165)	6 (−3;24)	14 (0;33)	5 (−3;18)	13 (0;29)	
LVESV [mL]								
All	63 (49;78)	64 (50;77)	61 (49;78)	0 (−6;8)	0 (−9;10)	0 (−9;14)	1 (−14;16)	
Empagliflozin	62 (49;76)	64 (51;75)	60 (49;75)	1 (−6;6)	0 (−6;11)	1 (−11;9)	−3 (−15;12)	**0.044**
Placebo	64 (49;80)	64 (48;82)	62 (49;79)	−1 (−9;7)	2 (−7;11)	0 (−7;18)	4 (−12;18)	
LVEF [%]	*n* = 244	*n* = 241	*n* = 230					
All	48 (43;52)	51 (47;56)	54 (48;58)	3 (−0;5)	5 (1;8)	5 (−1;12)	10 (2;18)	
Empagliflozin	48 (43;52)	52 (46;56)	55 (48;58)	3 (−0;6)	5 (1;9)	6 (−0;13)	11 (3;18)	0.888
Placebo	47 (43;52)	51 (47;55)	53 (49;56)	2 (−0;5)	5 (0;8)	4 (−1;11)	10 (1;18)	
LV−GLS [%]								
All	−16 (−19;−13)	−18 (−20;−15)	−19 (−21;−16)	−1 (−2;1)	−1 (−2;0)	10 (3;23)	16 (3;30)	
Empagliflozin	−16 (−19;−13)	−18 (−21;−16)	−19 (−22;−17)	−1 (−2;1)	−1 (−3;0)	10 (3;22)	15 (4;29)	0.728
Placebo	−15 (−19;−12)	−18 (−19;−15)	−18 (−21;−16)	−1 (−2;1)	−1 (−2;1)	10 (3;24)	18 (3;34)	
E/é								
All	9 (7;11)	8 (7;10)	8 (7;10)	1 (−4;7)	2 (−3;8)	−7 (−21;7)	−11 (−24;7)	
Empagliflozin	9 (7;11)	8 (7;10)	8 (6;9)	2 (−2;7)	2 (−3;8)	−8 (−21;9)	−11 (−25;7)	0.551
Placebo	9 (8;10)	8 (7;11)	8 (7;10)	0 (−6;7)	3 (−3;7)	−7 (−21;6)	−11 (−22;7)	
LAVI [mL/m^2^]								
All	31 (27;38)	32 (27;39)	33 (27;40)	−2 (−3;−1)	−3 (−4;−1)	1 (−13;19)	4 (−12;23)	
Empagliflozin	30 (26;37)	32 (26;39)	32 (27;39)	−2 (−4;−0)	−3 (−4;−1)	1 (−12;23)	3 (−12;23)	0.460
Placebo	32 (28;39)	32 (28;40)	34 (27;41)	−2 (−3;−1)	−3 (−5;−0)	1 (−13;17)	5 (−12;28)	
LA−GLS [%]								
All	19 (14;25)	22 (16;26)	22 (17;28)	0 (−8;5)	−2 (−9;6)	6 (−19;40)	12 (−16;46)	
Empagliflozin	19 (15;25)	22 (17;27)	22 (17;30)	−0 (−7;5)	−0 (−7;6)	9 (−11;41)	11 (−16;48)	0.098
Placebo	19 (14;26)	21 (15;26)	22 (16;28)	0 (−9;5)	−2 (−9;6)	3 (−25;38)	13 (−16;45)	
TAPSE [mm]								
All	21 (19;23)	23 (21;24)	23 (21;25)	−2 (−5;0)	−3 (−5;−1)	5 (−3;17)	10 (−1;20)	
Empagliflozin	21 (18;23)	23 (21;24)	23 (21;25)	−2 (−5;0)	−3 (−4;−1)	7 (−3;18)	11 (−3;21)	0.858
Placebo	21 (19;23)	22 (20;24)	23 (21;25)	−2 (−4;−0)	−3 (−5;−1)	5 (−3;16)	9 (1;20)	
RV−FAC [%]								
All	37 (34;42)	41 (37;45)	42 (39;46)	−2 (−6;1)	−3 (−6;−0)	7 (−1;18)	12 (2;23)	
Empagliflozin	37 (35;42)	41 (37;45)	43 (39;46)	−2 (−5;−0)	−3 (−7;−1)	7 (−4;17)	12 (2;24)	0.399
Placebo	38 (33;41)	42 (38;45)	42 (39;46)	−3 (−6;1)	−3 (−6;−0)	7 (1;18)	11 (2;22)	
RV−GLS [%]								
All	−21 (−23;−18)	−23 (−25;−21)	−24 (−26;−22)	3 (−0;6)	4 (1;8)	10 (−1;23)	14 (4;26)	
Empagliflozin	−21 (−23;−18)	−23 (−25;−21)	−24 (−26;−22)	2 (−2;6)	4 (1;9)	8 (−3;26)	12 (4;23)	0.197
Placebo	−21 (−23;−18)	−24 (−26;−21)	−24 (−26;−22)	3 (0;7)	4 (1;8)	11 (0;23)	14 (4;29)	
RV−FWS [%]								
All	−27 (−30;−24)	−29 (−32;−27)	−31 (−33;−28)	1 (−1;3)	2 (−0;4)	8 (−2;22)	10 (1;27)	
Empagliflozin	−27 (−30;−23)	−29 (−32;−27)	−31 (−33;−28)	1 (−1;4)	2 (−1;4)	9 (−5;26)	10 (0;25)	0.360
Placebo	−27 (−29;−24)	−30 (−32;−27)	−31 (−33;−28)	1 (−1;3)	2 (0;4)	7 (1;22)	11 (2;31)	
RAVI [mL/m^2^]								
All	23 (18;28)	23 (19;29)	24 (20;29)	0 (−4;6)	1 (−4;7)	4 (−13;27)	8 (−9;30)	
Empagliflozin	22 (18;27)	23 (19;27)	23 (20;29)	0 (−4;6)	1 (−4;6)	3 (−16;25)	8 (−14;30)	0.152
Placebo	23 (18;28)	25 (20;30)	25 (20;30)	0 (−5;5)	2 (−4;8)	5 (−9;29)	8 (−8;31)	
RA−GLS [%]								
All	35 (29;42)	35 (29;41)	34 (28;40)	1 (−3;5)	2 (−2;7)	0 (−21;17)	−4 (−22;19)	
Empagliflozin	35 (30;42)	36 (30;42)	35 (29;40)	1 (−4;4)	2 (−4;6)	0 (−17;16)	−1 (−20;19)	0.441
Placebo	35 (29;43)	34 (29;40)	33 (28;40)	1 (−2;6)	2 (−2;7)	1 (−21;17)	−5 (−22;18)	

All values reported in median (interquartile range). P-values reported from the linear mixed-effects model for average treatment effects, adjusted for baseline values, age, and sex. P-values meeting statistical significance are marked in bold. *LA-GLS* left-atrial global longitudinal strain, *LAVI* left-atrial volume index, *LVEDV* left-ventricular end-diastolic volume, *LVEF* left-ventricular ejection fraction, *LVESV* left-ventricular end-systolic volume, *LV-GLS* left-ventricular global longitudinal strain, *RA-GLS* right-atrial global longitudinal strain, *RAVI* right- atrial volume index, *RV-FAC* right-ventricular fractional area change, *RV-FWS* right-ventricular freewall strain, *RV-GLS* right-ventricular global longitudinal strain, *TAPSE* tricuspid annular plane systolic excursion

At week 6, an increase in LVEDV (5 [−3;18]%) and LAVI (1 [−13;19]%), and a roughly constant LVESV (0 [−9;14]%) was observed in the whole cohort. Parameters reflecting left ventricular, right ventricular, left atrial, and right atrial function including deformation analysis improved throughout the cohort (baseline to week 6: LVEF 5 [−1;12]%; LV-GLS 10 [3;23]%, E/e’ −7 [−21;7]%; LA-GLS 6 [−19;40]%; TAPSE 5 [−3;17]%; right-ventricular fractional area change [RV-FAC] 7 [−1;18]%; RV-GLS 10 [−1;23]%; RV-FWS 8 [−2;22]%; RA-GLS 0 [−21;17]%; RAVI 4 [−13;27]%).

**Fig. 2:**
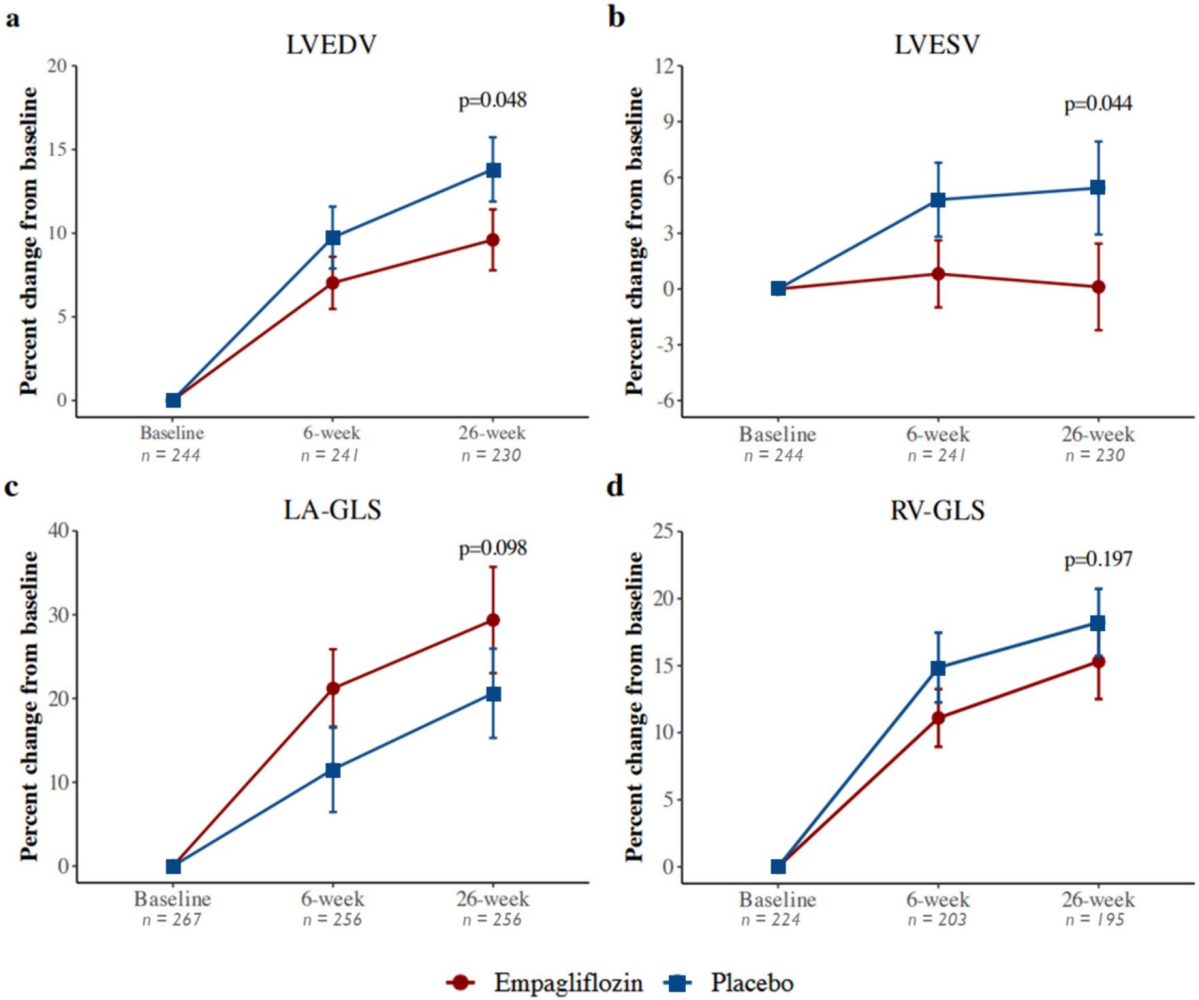
Changes in echocardiographic parameters by treatment group. **a** LVEDV: left-ventricular end-diastolic volume, **b** LVESV: left-ventricular end-systolic volume, **c** LA-GLS: left-atrial global longitudinal strain, and **d** RV-GLS: right-ventricular global longitudinal strain

At week 26, LVEDV and LAVI increased more pronounced in the placebo group, while LVESV decreased in the empagliflozin group (LVEDV 8 [-3;19]% with empagliflozin versus 13 [0;29]% with placebo, p = 0.048; LAVI 3 [−12;23]% vs. 5 [−12;28]%, p = 0.460; LVESV −3 [−15;12]% vs. 4 [−12;18]%, p = 0.044). This effect of empagliflozin on LVEDV and LVESV persisted after adjustment for baseline values, age, and sex between groups (Fig. 2A, B), in line with the findings of the main EMMY trial. [7]

Right ventricular functional parameters improved throughout the cohort (baseline to week 26: TAPSE 10 [−1;20]%; RV-FAC 12 [2;23]%; RV-GLS 14 [4;26]%; RV-FWS 10 [1;27]%). However, no statistically significant differences were observed between treatment groups (TAPSE p = 0.858; RV-FAC p = 0.399; RV-GLS p = 0.197; RV-FWS p = 0.360).

Parameters reflecting diastolic function, as depicted by E/e’, LA-GLS, and LAVI showed a distinct trend over the course of the trial when comparing groups but did not meet statistical significance. (Fig. 2 and Figure S2).

### Reproducibility

Each echocardiographic measurement was performed on two separate cardiac cycles, if applicable, and reported as mean values. Parameters showed overall good reproducibility. The intraclass correlation coefficients are provided in the Online Resource Table S2.

## Discussion

Among the 301 patients analyzed in this sub-study of the EMMY trial with in-depth echocardiographic analysis performed at baseline, 6 weeks, and 26 weeks after an AMI, randomization to empagliflozin resulted in a significant improvement of left ventricular volumes compared to a placebo. These observations suggest a primary effect of empagliflozin in terms of mitigated cardiac remodeling after AMI (Tables 2 and S3).

Moreover, this echocardiographic post-hoc sub-analysis is the first study to assess the trajectories of non-left ventricular deformation analysis after AMI. However, regarding right ventricular function and strain analysis, no differences were observed between treatment groups.

Though new advances in interventions and pharmacologic treatments after AMI are emerging, it remains one of the most important causes of morbidity and mortality worldwide [17]. Cardiac remodeling after AMI, characterized by fibrosis, chamber dilation, and dysfunction, is a major risk factor in disease development and is modifiable with pharmacologic agents like ACEi, ARB, ARNI, and beta-blockers. These agents have shown significant effects on remodeling in patients with heart failure as well as post-AMI, which in a longer-term has a major impact on relevant clinical outcomes [1–4, 18, 19].

Reversing remodeling plays a crucial role in reducing morbidity and mortality in patients after AMI [20]. Like in the field of heart failure, accumulating evidence points to beneficial effects of SGLT2i in patients with coronary artery disease and acute coronary syndromes, beyond their glucose-lowering properties [3, 7, 21–24]. In the previously published dapagliflozin in patients with myocardial infarction (DAPA-MI) trial, the administration of dapagliflozin in addition to standard post-MI therapy after AMI demonstrated a favorable effect on cardiometabolic outcomes compared to a placebo in patients without diabetes or chronic heart failure, despite failing to show significant differences regarding a composite endpoint of cardiovascular death and hospitalization for heart failure [25]. Similar neutral effects of empagliflozin on prespecified clinical outcomes were observed in the empagliflozin in patients post myocardial infarction (EMPACT-MI) trial, however, a sub-analysis showed a risk reduction for first and recurrent heart failure hospitalizations with empagliflozin [26, 27]. Overall there seems to be a positive effect of SGLT2i on the cardiovascular system after AMI. Underlying pathways on the prevention and reversal of adverse cardiac remodeling are widely discussed and deemed multifactorial. Several preclinical and clinical studies suggest the anti-inflammatory and anti-fibrotic properties[28–30] as well as improved cardiac efficiency and increased myocardial energy supply [31, 32] to contribute to their cardioprotective benefits.

This echocardiographic post-hoc analysis demonstrates significant effects on cardiac remodeling depicted by changes in left ventricular diastolic and systolic volumes in favor of the empagliflozin group, which confirms the findings of the main EMMY trial [7]. On the other hand, only favorable trends but no significant effect was observed for markers of left-ventricular systolic and diastolic function. On regard of this, similar trajectories were observed in left atrial, right ventricular, and right atrial parameters, and throughout deformation analysis parameters with and without empagliflozin. The overall baseline LVEF and LV-GLS was only mildly reduced in this study. Especially in cohorts with less impaired systolic function, the clinical effect on cardiac function after AMI seems limited. The SOCOGAMI trial demonstrated a significant decrease in body weight and blood glucose but showed no significant influence of empagliflozin on echocardiographic and magnetic resonance imaging variables in a cohort with recent ACS and normal LV function [33]. This, in alignment with the results of the present study, poses the question of treatment effect in a subset of patients after AMI with more pronounced functional impairment. However, the SUGAR-DM-HF trial examined structural changes in chronic heart failure with reduced ejection fraction (HFrEF) in patients with type 2 diabetes treated with empagliflozin and demonstrated a reduction of diastolic and systolic left ventricular volumes with no changes of systolic function [22]. In the EMPA-VISION trial patients with either HFrEF or HFpEF were examined using cardiac magnetic resonance. Here, though not reaching statistical significance, a favorable trend towards empagliflozin was observed regarding left ventricular strain, which was more pronounced in the HFpEF group (adjusted mean treatment difference, 2.18% [SE, 1.16 (95% CI, − 0.28 to 4.64)]; p = 0.08) [34].

Of note, available recent echocardiographic sub-studies of trials investigating cardiovascular outcomes after AMI for sacubitril/valsartan and vericiguat also showed only minor effects on cardiac structure, and mostly no effect on cardiac function. The echocardiographic sub-study of the PARADISE-MI trial, which investigated the impact of ARNI versus ACEi in 544 patients after AMI, showed significant change in LVEDV in the ARNI group (delta ARNI 0 ± 29 mL vs. ACEi 5 ± 30 mL, p = 0.025) but no significant differences in LVEF improvement (delta ARNI 5.4 ± 9.5% vs. ACEi 6.6 ± 10.7%, p = 0.79) [3]. In the VICTORIA echocardiographic sub-study, comparing vericiguat versus placebo in 419 patients with HFrEF with a recent worsening heart failure event, significant changes could only be demonstrated for left ventricular volumes (delta LVEDVi −2.9 ± 18.5 mL vs. −7.7 ± 23.7 mL, p = 0.021) and not left ventricular systolic function (delta LVEF vericiguat 3.2 ± 8.0% vs. placebo 2.4 ± 7.6%, p = 0.091) [35].

### Strengths and limitations

The EMMY trial was the first trial to show the effect of early SGLT2i therapy in patients after AMI, predominantly in patients without diabetes. This post-hoc analysis provides first insight into cardiac mechanisms investigated with echocardiography in the first 26 weeks of treatment compared with placebo. Overall, the data in this study reflects the data presented in the main EMMY trial, however, parameters reflecting systolic and diastolic function, in contrast to the analysis in the main trial, did not meet significance in this sub-cohort, most probably due to lack of statistical power (post-hoc power estimation at an alpha level of 0.05: LVEF 43%, E/e’ 60%).

Several considerations are relevant regarding these deviating results. This post-hoc analysis was based on a significantly smaller sample size, with only three participating sites compared to 11 in the main trial. Moreover, a relevant portion of echocardiographic examinations did not meet the criteria for post-processing analysis and had to be left out of the final analyses. Especially deformation imaging is highly dependent on image quality, therefore the ability to discern a treatment-related difference with empagliflozin may have been mitigated by the limited sample size.

On the other hand, all analyses were conducted at the Central Echocardiographic Core Laboratory by trained, blinded investigators, twice if applicable, with an overall satisfying intra-observer reproducibility. This enabled the acquisition of a comprehensive dataset with precise measurements. Furthermore, to our best knowledge, this is the first study to give insight to the course of cardiac structure and function including right ventricular and atrial structural and functional parameters including deformation imaging after AMI.

However, our findings may have a limited application in women, as only 18.3% of the study population was female. This underrepresentation of women is a common finding in cardiovascular trials, particularly in AMI trials [36, 37]. Some of the inclusion criteria of the EMMY trial may have contributed to the smaller number of females. For inclusion, evidence of high cardiac biomarker levels was needed, but literature shows that in women these levels are lower compared to men [38]. However, a sub-analysis of the EMMY trial indicated no significant difference in treatment effect between genders [39].

## Conclusion

Among patients with recent acute myocardial infarction, the early initiation of empagliflozin after PCI resulted in a significant effect on left ventricular volumes compared to a placebo after 26 weeks. Parameters reflecting diastolic function (E/e’, LA-GLS, LAVI) and parameters of the right ventricle (TAPSE, RV-FAC, RV-GLS, RV-FWS) showed a beneficial trend, but did not meet significance in this cohort.

## Supplementary Information

Below is the link to the electronic supplementary material.

Supplementary file1 (DOCX 557 KB)

## Data Availability

The data underlying this article will be shared on reasonable request to the corresponding author.

## Abbreviations

LVEDV: Left ventricular end-diastolic volume
LVESV: Left ventricular end-systolic volume
RCT: Randomized controlled trial
TTE: Transthoracic echocardiography
ULN: Upper limit of normal
