# Supplementary material for: Impact of empagliflozin on cardiac structure and function assessed by echocardiography after myocardial infarction: a post-hoc sub-analysis of the emmy trial
Source: Clin Res Cardiol. 2024 Sep 16;114(5):629–39. doi: 10.1007/s00392-024-02523-1 (PMC12058928; doi:10.1007/s00392-024-02523-1)

**Supplemental Material**

**Impact of Empagliflozin on Cardiac Structure and Function assessed by Echocardiography after Myocardial Infarction: a post-hoc sub-analysis of the EMMY trial**

*Clinical Research in Cardiology*

Nora Schwegel^1^, Christoph Strohhofer^1^, Ewald Kolesnik^1,#^, Sabrina Oltean^1^, Alexander Hüttmair^1^, Christian Pipp^1^, Martin Benedikt^1^, Nicolas Verheyen^1^, Johannes Gollmer^1^, Klemens Ablasser^1^, Markus Wallner^1^, Viktoria Santner^1^, Norbert Tripolt^2^, Peter Pferschy^2^, Peter Zechner^3^, Hannes Alber^4^, Jolanta M. Siller-Matula^5^, Kristen Kopp^6^, Andreas Zirlik^1^, Faisal Aziz^2^, Harald Sourij^2,*^, and Dirk von Lewinski^1,*^

*1 Division of Cardiology, University Heart Center Graz, Medical University of Graz, Graz, Austria*

*2 Trials Unit for Interdisciplinary Metabolic Medicine, Division of Endocrinology and Diabetology, Department of Internal Medicine, Medical University of Graz, Graz, Austria*

*3 Department of Cardiology and Intensive Care Medicine, Hospital Graz II, West Location, Graz, Austria*

*4 Department of Cardiology, Public Hospital Klagenfurt am Woerthersee, Klagenfurt am Woerthersee, Austria*

*5 Department of Cardiology, Medical University of Vienna, Vienna, Austria*

*6 Division of Cardiology and Internal Intensive Care Medicine, Department of Internal Medicine II, Paracelsus Medical Private University of Salzburg, Salzburg, Austria*

*# corresponding author*

** contributed equally*

**supplemental Tables**

**Table S1** Echocardiographic measurements conducted in the echocardiographic post-hoc sub-analysis of the EMMY trial

|  | **Parameter** | **Method** |
| --- | --- | --- |
| **Left ventricle** | Intraventricular septum thickness (IVSed) | Assessed in the parasternal long axis at level of the mitral leaflet tips in end diastole |
|  | Posterior wall thickness (PWed) |  |
|  | End-diastolic diameter (LVEDD) |  |
|  | End-diastolic volume (EDV) biplane | Biplane Simpson’s method in apical four- and two-chamber view |
|  | End-systolic volume (ESV) biplane |  |
|  | Ejection fraction (EF) biplane |  |
|  | EDV triplane | Calculated as mean of corresponding volumes derived from disc summation method in apical four-, three-, and two-chamber views using TomTec 2DCPA* |
|  | ESV triplane |  |
|  | EF triplane |  |
|  | LV-Global longitudinal strain (GLS) | Assessed in apical four-, three-, and two-chamber views using TomTec 2DCPA*; calculated from the entire endocardial contour line length in a 16-segment model |
|  | Autostrain | Assessed in apical four-, three-, and two-chamber views using the automated strain measurement tool from TomTec*; calculated from the entire endocardial contour line length |
|  | e‘ lateral (e’_lat_) | Tissue Doppler derived peak early diastolic tissue velocity (e’) measured from the lateral and septal aspect of the mitral annulus |
|  | e‘ septal (e‘_sep_) |  |
|  | E | Mitral inflow velocity (E) derived from pulsed-wave Doppler |
|  | E/e‘ | Calculated from E and e’ (averaged e’_lat_ and e’_sep_) |
| **Left atrium** | Diameter | Parasternal long axis, assessed in end-systole at the level of the aortic root |
|  | Left atrial volume (LAV) | Biplane Simpson’s method in apical four- and two-chamber views in end-systole |
|  | LAV-Index (LAVI) | LAV indexed to body surface area |
|  | LA-GLS | Assessed in apical two-chamber view to avoid impairment by pulmonary veins using TomTec 2DCPA*; calculated from the entire endocardial contour line length |
| **Right ventricle** | End-diastolic diameter (EDD) basal | Assessed in RV-focused apical four-chamber view in end-diastole at RV base (EDD basal), mid-ventricular (EDD mid), and in the longitudinal axis (ED length) |
|  | EDD mid |  |
|  | ED length |  |
|  | ED Area | Measured in RV-focused apical four-chamber view in end-diastole |
|  | TAPSE | Measured in M-mode placed through the lateral tricuspid anulus |
|  | RV-GLS | Assessed in RV-focused apical four-chamber view by averaging longitudinal systolic strain values of the entire endocardial contour line length of septal and free wall segments (six segment model, RVGLS) and from the free wall segments alone (three segment model, RVFWS); using TomTec 2DCPA* |
|  | Freewall Strain (RV-FWS) | Assessed in RV-focused apical four-chamber view by averaging longitudinal systolic strain values of the entire endocardial contour line length of septal and freewall segments (six segment model, RVGLS) and from the  freewall segments alone (three segment model, RVFWS); using TomTec 2DCPA* |
|  | s‘ lateral | Derived from pulsed-wave Doppler with active tissue Doppler (spectral Doppler) placed on the lateral tricuspid annulus |
|  | Tricuspid valve closure opening time (TCO) | Derived from pulsed-wave Doppler with active tissue Doppler (spectral Doppler) placed on the lateral tricuspid annulus  Calculated using TCO and ET |
|  | Ejection time (ET) |  |
|  | Tei Index |  |
| **Right atrium** | Right atrial volume (RAV) | Biplane Simpson’s method in apical four-chamber view |
|  | RAV-Index (RAVI) | RAV indexed to body surface area |
|  | RA-GLS | Assessed in apical four-chamber view using TomTec 2DCPA*; calculated from the entire endocardial contour line length |
|  | Peak tricuspid regurgitation velocity (TRVmax) | Measured from continuous-wave spectral Doppler envelope |
|  | Right atrial pressure (RAP) | Estimated from diameter and collapse of the inferior vena cava (IVC) during respiration; if IVC ≤21mm that collapses >50% RAP is estimated with 5mmHg; if IVC >21mm that collapses <50% RAP is estimated with 15mmHg; in indeterminate cases a RAP of 10mmHg was used |
|  | Systolic pulmonary artery pressure (sPAP) | Calculated from TRVmax and estimated RAP |

Assessment of parameters according to current ESC/AHA/ERS guidelines.^12-18^ *TomTec 2-dimensional Cardiac Performance Analysis.

**Table S2** Intra-observer reproducibility metrics

|  | **Baseline** | | | **Week 6** | | | **Week 26** | | |
| --- | --- | --- | --- | --- | --- | --- | --- | --- | --- |
|  | **Bias ± SD** | **ICC (95%CI)** | **p-value** | **Bias ± SD** | **ICC (95%CI)** | **p-value** | **Bias ± SD** | **ICC (95%CI)** | **p-value** |
| **Left ventricle** |  |  |  |  |  |  |  |  |  |
| EDV biplane | 5.14±5.54mL | 0.986 (0.981-0.989) | <0.001 | 5.78±6.53mL | 0.983 (0.978-0.987) | <0.001 | 5.25±6.36mL | 0.987 (0.983-0.990) | <0.001 |
| ESV biplane | 3.17±3.96mL | 0.986 (0.982-0.990) | <0.001 | 3.16±3.67mL | 0.989 (0.986-0.992) | <0.001 | 3.05±3.93mL | 0.990 (0.987-0.992) | <0.001 |
| EF biplane | 1.33±1.53% | 0.979 (0.972-0.983) | <0.001 | 1.19±1.23% | 0.986 (0.982-0.989) | <0.001 | 1.24±1.49% | 0.984 (0.979-0.988) | <0.001 |
| EDV triplane | 6.37±7.20mL | 0.975 (0.967-0.981) | <0.001 | 5.52±5.74mL | 0.987 (0.982-0.990) | <0.001 | 5.62±6.12mL | 0.987 (0.983-0.990) | <0.001 |
| ESV triplane | 4.19±5.03mL | 0.976 (0.969-0.982) | <0.001 | 3.60±4.25mL | 0.986 (0.982-0.989) | <0.001 | 3.26±3.40mL | 0.992 (0.989-0.994) | <0.001 |
| EF triplane | 1.85±2.12% | 0.962 (0.950-0.971) | <0.001 | 1.77±2.02% | 0.967 (0.957-0.975) | <0.001 | 1.74±1.95% | 0.972 (0.963-0.978) | <0.001 |
| LV-GLS | 0.86±0.86% | 0.978 (0.971-0.983) | <0.001 | 0.88±0.91% | 0.972 (0.963-0.979) | <0.001 | 0.89±0.90% | 0.971 (0.962-0.978) | <0.001 |
| Autostrain | 0.54±0.53% | 0.990 (0.987-0.992) | <0.001 | 0.52±0.54% | 0.989 (0.986-0.992) | <0.001 | 0.45±0.42% | 0.993 (0.990-0.994) | <0.001 |
| **Left atrium** |  |  |  |  |  |  |  |  |  |
| LAV | 5.20±5.27mL | 0.963 (0.952-0.971) | <0.001 | 5.34±4.47mL | 0.969 (0.960-0.976) | <0.001 | 5.72±6.72mL | 0.958 (0.946-0.967) | <0.001 |
| LA-GLS | 5.17±4.80% | 0.798 (0.743-0.842) | <0.001 | 5.35±4.50% | 0.830 (0.781-0.868) | <0.001 | 5.13±4.51% | 0.842 (0.797-0.877) | <0.001 |
| **Right ventricle** |  |  |  |  |  |  |  |  |  |
| End-diastolic area | 0.71±0.69cm^2^ | 0.987 (0.984-0.990) | <0.001 | 0.82±1.07cm^2^ | 0.977 (0.970-0.982) | <0.001 | 0.71±0.59cm^2^ | 0.989 (0.986-0.992) | <0.001 |
| RV-GLS | 2.56±2.12% | 0.833 (0.781-0.872) | <0.001 | 2.12±1.68% | 0.844 (0.792-0.883) | <0.001 | 2.06±1.76% | 0.848 (0.797-0.886) | <0.001 |
| RV-FWS | 3.26±2.94% | 0.814 (0.757-0.858) | <0.001 | 2.87±2.24% | 0.829 (0.771-0.871) | <0.001 | 2.62±2.26% | 0.852 (0.802-0.889) | <0.001 |
| **Right atrium** |  |  |  |  |  |  |  |  |  |
| RAV | 2.85±2.77mL | 0.986 (0.981-0.990) | <0.001 | 2.77±2.42mL | 0.989 (0.984-0.992) | <0.001 | 3.02±3.09mL | 0.985 (0.981-0.989) | <0.001 |
| RA-GLS | 6.91±6.16% | 0.773 (0.709-0.822) | <0.001 | 6.96±5.73% | 0.754 (0.685-0.808) | <0.001 | 6.82±5.48% | 0.783 (0.721-0.832) | <0.001 |

Conducted in the whole cohort. Bias ± standard deviation (SD), intra-class correlation coefficient (ICC) with 95% confidence interval (CI), and according p-value. *GLS, global longitudinal strain; EDV, end-diastolic volume; ESV, end-systolic volume; EF, ejection fraction; RV-FWS, right ventricular freewall strain; LAV, left atrial volume; RAV, right atrial volume*.

**Table S3** Echocardiographic parameters per visit

|  | **Baseline** | **Week 6** | **Week 26** |
| --- | --- | --- | --- |
| **IVSed** *[mm]* |  |  |  |
| All | 12 (11;13) | 11 (10;12) | 11 (10;12) |
| Empagliflozin | 12 (11;13) | 11 (10;12) | 11 (10;12) |
| Placebo | 12 (11;13) | 11 (10;12) | 11 (10;12) |
| **PWed** *[mm]* |  |  |  |
| All | 11 (10;12) | 11 (10;12) | 10 (10;11) |
| Empagliflozin | 11 (10;12) | 11 (10;12) | 10 (10;11) |
| Placebo | 11 (10;13) | 11 (10;12) | 10 (10;11) |
| **LVEDDbasal biplane** *[mm]* |  |  |  |
| All | 50 (47;53) | 52 (48;55) | 51 (48;55) |
| Empagliflozin | 50 (47;53) | 52 (48;55) | 51 (48;55) |
| Placebo | 50 (46;54) | 52 (48;55) | 52 (48;55) |
| **LVEDDbasal triplane** *[mm]* |  |  |  |
| All | 4 (3;4) | 4 (3;4) | 4 (3;4) |
| Empagliflozin | 4 (3;4) | 4 (3;4) | 4 (3;4) |
| Placebo | 4 (3;4) | 4 (3;4) | 4 (3;4) |
| **LVEDV biplane** *[mL]* |  |  |  |
| All | 122 (100;142) | 132 (110;151) | 135 (114;158) |
| Empagliflozin | 120 (100;140) | 132 (113;149) | 135 (114;155) |
| Placebo | 122 (101;145) | 132 (107;155) | 134 (111;165) |
| **LVESV biplane** *[mL]* |  |  |  |
| All | 63 (49;78) | 64 (50;77) | 61 (49;78) |
| Empagliflozin | 62 (49;76) | 64 (51;75) | 60 (49;75) |
| Placebo | 64 (49;80) | 64 (48;82) | 62 (49;79) |
| **LVEF biplane** *[%]* |  |  |  |
| All | 48 (43;52) | 51 (47;56) | 54 (48;58) |
| Empagliflozin | 48 (43;52) | 52 (46;56) | 55 (48;58) |
| Placebo | 47 (43;52) | 51 (47;55) | 53 (49;56) |
| **LVEDV triplane** *[mL]* |  |  |  |
| All | 118 (99;138) | 130 (106;153) | 131 (112;152) |
| Empagliflozin | 116 (97;138) | 130 (112;149) | 131 (113;148) |
| Placebo | 119 (101;138) | 130 (104;154) | 131 (110;157) |
| **LVESV triplane** *[mL]* |  |  |  |
| All | 61 (49;75) | 63 (49;77) | 61 (49;78) |
| Empagliflozin | 60 (49;73) | 63 (50;76) | 61 (50;77) |
| Placebo | 64 (51;76) | 64 (48;79) | 61 (47;78) |
| **LVEF triplane** *[%]* |  |  |  |
| All | 49 (43;53) | 51 (47;56) | 54 (48;58) |
| Empagliflozin | 49 (43;53) | 52 (47;57) | 55 (46;58) |
| Placebo | 49 (42;54) | 51 (47;56) | 53 (48;57) |
| **LV-GLS** *[%]* |  |  |  |
| All | -16 (-19;-13) | -18 (-20;-15) | -19 (-21;-16) |
| Empagliflozin | -16 (-19;-13) | -18 (-21;-16) | -19 (-22;-17) |
| Placebo | -15 (-19;-12) | -18 (-19;-15) | -18 (-21;-16) |
| **Autostrain** *[%]* |  |  |  |
| All | -16 (-19;-13) | -18 (-20;-16) | -19 (-21;-17) |
| Empagliflozin | -17 (-19;-14) | -19 (-21;-16) | -20 (-22;-17) |
| Placebo | -16 (-19;-13) | -18 (-20;-16) | -19 (-21;-17) |
| **e' lat** |  |  |  |
| All | 8 (7;10) | 9 (7;11) | 9 (7;11) |
| Empagliflozin | 9 (7;11) | 9 (8;11) | 10 (8;12) |
| Placebo | 8 (6;10) | 9 (7;11) | 8 (7;11) |
| **e'sep** |  |  |  |
| All | 7 (6;9) | 7 (6;8) | 7 (6;8) |
| Empagliflozin | 7 (6;9) | 7 (6;8) | 7 (6;8) |
| Placebo | 7 (6;8) | 7 (6;8) | 7 (6;8) |

|  | **Baseline** | **Week 6** | **Week 26** |
| --- | --- | --- | --- |
| **E** |  |  |  |
| All | 71 (59;82) | 67 (56;78) | 66 (56;77) |
| Empagliflozin | 72 (60;83) | 67 (57;79) | 66 (56;77) |
| Placebo | 70 (58;81) | 66 (54;76) | 65 (57;77) |
| **E/e'** |  |  |  |
| All | 9 (7;11) | 8 (7;10) | 8 (7;10) |
| Empagliflozin | 9 (7;11) | 8 (7;10) | 8 (6;9) |
| Placebo | 9 (8;10) | 8 (7;11) | 8 (7;10) |
| **LA diameter** *[mm]* |  |  |  |
| All | 39 (36;43) | 40 (37;44) | 40 (36;44) |
| Empagliflozin | 39 (35;43) | 40 (37;44) | 40 (36;43) |
| Placebo | 39 (37;43) | 41 (37;45) | 41 (37;45) |
| **LAV** *[mL]* |  |  |  |
| All | 63 (51;74) | 64 (52;78) | 65 (53;82) |
| Empagliflozin | 61 (50;72) | 64 (51;75) | 64 (52;78) |
| Placebo | 65 (53;78) | 64 (53;81) | 67 (55;84) |
| **LAVI** *[mL/m^2^]* |  |  |  |
| All | 31 (27;38) | 32 (27;39) | 33 (27;40) |
| Empagliflozin | 30 (26;37) | 32 (26;39) | 32 (27;39) |
| Placebo | 32 (28;39) | 32 (28;40) | 34 (27;41) |
| **LA-GLS** *[%]* |  |  |  |
| All | 19 (14;25) | 22 (16;26) | 22 (17;28) |
| Empagliflozin | 19 (15;25) | 22 (17;27) | 22 (17;30) |
| Placebo | 19 (14;26) | 21 (15;26) | 22 (16;28) |
| **RV EDD basal** *[mm]* |  |  |  |
| All | 36 (33;40) | 38 (34;41) | 37 (34;41) |
| Empagliflozin | 36 (34;40) | 38 (34;40) | 37 (34;40) |
| Placebo | 37 (33;41) | 38 (35;41) | 38 (35;42) |
| **RV EDD mid** *[mm]* |  |  |  |
| All | 27 (24;30) | 28 (25;31) | 29 (26;32) |
| Empagliflozin | 28 (24;30) | 28 (25;31) | 28 (25;31) |
| Placebo | 27 (24;30) | 28 (25;31) | 29 (26;32) |
| **RV ED length** *[mm]* |  |  |  |
| All | 78 (73;85) | 79 (74;83) | 79 (74;84) |
| Empagliflozin | 77 (73;85) | 79 (73;83) | 79 (74;84) |
| Placebo | 79 (73;84) | 79 (74;83) | 80 (74;84) |
| **RV ED area** *[cm^2^]* |  |  |  |
| All | 20 (18;23) | 21 (18;24) | 21 (18;24) |
| Empagliflozin | 20 (18;22) | 21 (18;23) | 21 (18;24) |
| Placebo | 20 (18;23) | 21 (18;24) | 21 (18;24) |
| **TAPSE** *[mm]* |  |  |  |
| All | 21 (19;23) | 23 (21;24) | 23 (21;25) |
| Empagliflozin | 21 (18;23) | 23 (21;24) | 23 (21;25) |
| Placebo | 21 (19;23) | 22 (20;24) | 23 (21;25) |
| **RV-FAC** *[%]* |  |  |  |
| All | 37 (34;42) | 41 (37;45) | 42 (39;46) |
| Empagliflozin | 37 (35;42) | 41 (37;45) | 43 (39;46) |
| Placebo | 38 (33;41) | 42 (38;45) | 42 (39;46) |
| **RV-GLS** *[%]* |  |  |  |
| All | -21 (-23;-18) | -23 (-25;-21) | -24 (-26;-22) |
| Empagliflozin | -21 (-23;-18) | -23 (-25;-21) | -24 (-26;-22) |
| Placebo | -21 (-23;-18) | -24 (-26;-21) | -24 (-26;-22) |
| **RV-FWS** *[%]* |  |  |  |
| All | -27 (-30;-24) | -29 (-32;-27) | -31 (-33;-28) |
| Empagliflozin | -27 (-30;-23) | -29 (-32;-27) | -31 (-33;-28) |
| Placebo | -27 (-29;-24) | -30 (-32;-27) | -31 (-33;-28) |
| **s' lateral** *[cm/sec]* |  |  |  |
| All | 10 (9;12) | 10 (9;11) | 10 (9;11) |
| Empagliflozin | 10 (9;12) | 10 (9;11) | 10 (9;11) |
| Placebo | 10 (9;12) | 10 (9;11) | 10 (9;11) |
|  | **Baseline** | **Week 6** | **Week 26** |
| **TCO** *[ms]* |  |  |  |
| All | 424 (394;454) | 458 (435;490) | 476 (445;504) |
| Empagliflozin | 426 (386;456) | 459 (432;494) | 480 (446;506) |
| Placebo | 420 (397;454) | 458 (436;486) | 470 (442;499) |
| **ET** *[ms]* |  |  |  |
| All | 274 (252;298) | 306 (284;329) | 318 (296;339) |
| Empagliflozin | 275 (252;298) | 308 (288;330) | 318 (298;339) |
| Placebo | 274 (252;296) | 304 (279;326) | 316 (294;340) |
| **Tei Index** |  |  |  |
| All | 1 (0;1) | 0 (0;1) | 0 (0;1) |
| Empagliflozin | 1 (0;1) | 0 (0;1) | 1 (0;1) |
| Placebo | 1 (0;1) | 0 (0;1) | 0 (0;1) |
| **RAV** *[mL]* |  |  |  |
| All | 44 (36;55) | 47 (37;57) | 49 (38;59) |
| Empagliflozin | 44 (36;54) | 46 (37;56) | 49 (38;58) |
| Placebo | 45 (35;57) | 49 (39;62) | 50 (39;60) |
| **RAVI** *[mL/m^2^]* |  |  |  |
| All | 23 (18;28) | 23 (19;29) | 24 (20;29) |
| Empagliflozin | 22 (18;27) | 23 (19;27) | 23 (20;29) |
| Placebo | 23 (18;28) | 25 (20;30) | 25 (20;30) |
| **RA-GLS** *[%]* |  |  |  |
| All | 35 (29;42) | 35 (29;41) | 34 (28;40) |
| Empagliflozin | 35 (30;42) | 36 (30;42) | 35 (29;40) |
| Placebo | 35 (29;43) | 34 (29;40) | 33 (28;40) |
| **TRVmax** *[m/sec]* |  |  |  |
| All | 2 (2;3) | 2 (2;3) | 2 (2;3) |
| Empagliflozin | 2 (2;3) | 2 (2;3) | 2 (2;3) |
| Placebo | 2 (2;3) | 2 (2;3) | 2 (2;3) |
| **sPAP** *[mmHg]* |  |  |  |
| All | 30 (26;34) | 28 (24;32) | 28 (23;32) |
| Empagliflozin | 29 (24;32) | 29 (26;32) | 28 (23;32) |
| Placebo | 30 (26;34) | 27 (24;32) | 28 (23;32) |

All values reported in median (interquartile range). *ET, ejection time; e’lat, e’ lateral; e’sep, e’ septal; GLS, global longitudinal strain; IVSed, intraventricular septum thickness in end-diastole; LAV, left-atrial volume; LAVI, left-atrial volume index; LVEDDbasal, left-ventricular end-diastolic diameter basal; LVEDV, left-ventricular end-diastolic volume; LVEF, left-ventricular ejection fraction; LVESV, left-ventricular end-systolic volume; RAV, right-atrial volume; RAVI, right-atrial volume index; RVEDD basal, right-ventricular end-diastolic diameter basal; RVEDD mid, right-ventricular end-diastolic diameter mid-ventricular; RV-FAC, right-ventricular fractional area change; RV-FWS, right-ventricular freewall strain; sPAP, systolic pulmonary artery pressure; TAPSE, tricuspid annular plane systolic excursion; TCO, tricuspid closure-open time; TRVmax, peak tricuspid regurgitation velocity; PWed, posterior wall thickness in end-diastole.*

**Table S4** Available measurements of in depth echocardiographic parameters per visit

|  | **Baseline** | **Week 6** | **Week 26** |
| --- | --- | --- | --- |
| **IVSed** *[n]* | *299* | *274* | *270* |
| **PWed** *[n]* | *292* | *274* | *270* |
| **LVEDDbasal biplane** *[n]* | *292* | *274* | *270* |
| **LVEDDbasal triplane** *[n]* | *233* | *227* | *220* |
| **LVEDV biplane** *[n]* | *244* | *241* | *230* |
| **LVESV biplane** *[n]* | *244* | *241* | *230* |
| **LVEF biplane** *[n]* | *244* | *241* | *230* |
| **LVEDV triplane** *[n]* | *233* | *227* | *220* |
| **LVESV triplane** *[n]* | *233* | *227* | *220* |
| **LVEF triplane** *[n]* | *233* | *227* | *220* |
| **LV-GLS** *[n]* | *233* | *227* | *220* |
| **Autostrain** *[n]* | *225* | *221* | *220* |
| **e' lat** *[n]* | *257* | *264* | *257* |
| **e'sep** *[n]* | *278* | *266* | *261* |
| **E** *[n]* | *288* | *276* | *272* |
| **E/e'** *[n]* | *257* | *264* | *257* |
| **LA diameter** *[n]* | *276* | *253* | *247* |
| **LAV** *[n]* | *254* | *250* | *238* |
| **LAVI** *[n]* | *254* | *250* | *238* |
| **LA-GLS** *[n]* | *267* | *256* | *256* |
| **RV EDD basal** *[n]* | *248* | *234* | *235* |
| **RV EDD mid** *[n]* | *248* | *234* | *235* |
| **RV ED length** *[n]* | *248* | *234* | *235* |
| **RV ED area** *[n]* | *248* | *234* | *235* |
| **TAPSE** *[n]* | *256* | *255* | *254* |
| **RV-FAC** *[n]* | *242* | *229* | *229* |
| **RV-GLS** *[n]* | *224* | *203* | *195* |
| **RV-FWS** *[n]* | *224* | *203* | *195* |
| **s' lateral** *[n]* | *203* | *192* | *196* |
| **TCO** *[n]* | *202* | *192* | *196* |
| **ET** *[n]* | *202* | *192* | *196* |
| **Tei Index** *[n]* | *202* | *192* | *196* |
| **RAV** *[n]* | *264* | *257* | *252* |
| **RAVI** *[n]* | *264* | *257* | *252* |
| **RA-GLS** *[n]* | *262* | *254* | *244* |
| **TRVmax** *[n]* | *164* | *160* | *167* |
| **sPAP** *[n]* | *157* | *155* | *165* |

*ET, ejection time; e’lat, e’ lateral; e’sep, e’ septal; GLS, global longitudinal strain; IVSed, intraventricular septum thickness in end-diastole; LAV, left-atrial volume; LAVI, left-atrial volume index; LVEDDbasal, left-ventricular end-diastolic diameter basal; LVEDV, left-ventricular end-diastolic volume; LVEF, left-ventricular ejection fraction; LVESV, left-ventricular end-systolic volume; RAV, right-atrial volume; RAVI, right-atrial volume index; RVEDD basal, right-ventricular end-diastolic diameter basal; RVEDD mid, right-ventricular end-diastolic diameter mid-ventricular; RV-FAC, right-ventricular fractional area change; RV-FWS, right-ventricular freewall strain; sPAP, systolic pulmonary artery pressure; TAPSE, tricuspid annular plane systolic excursion; TCO, tricuspid closure-open time; TRVmax, peak tricuspid regurgitation velocity; PWed, posterior wall thickness in end-diastole.*

**supplemental figures**


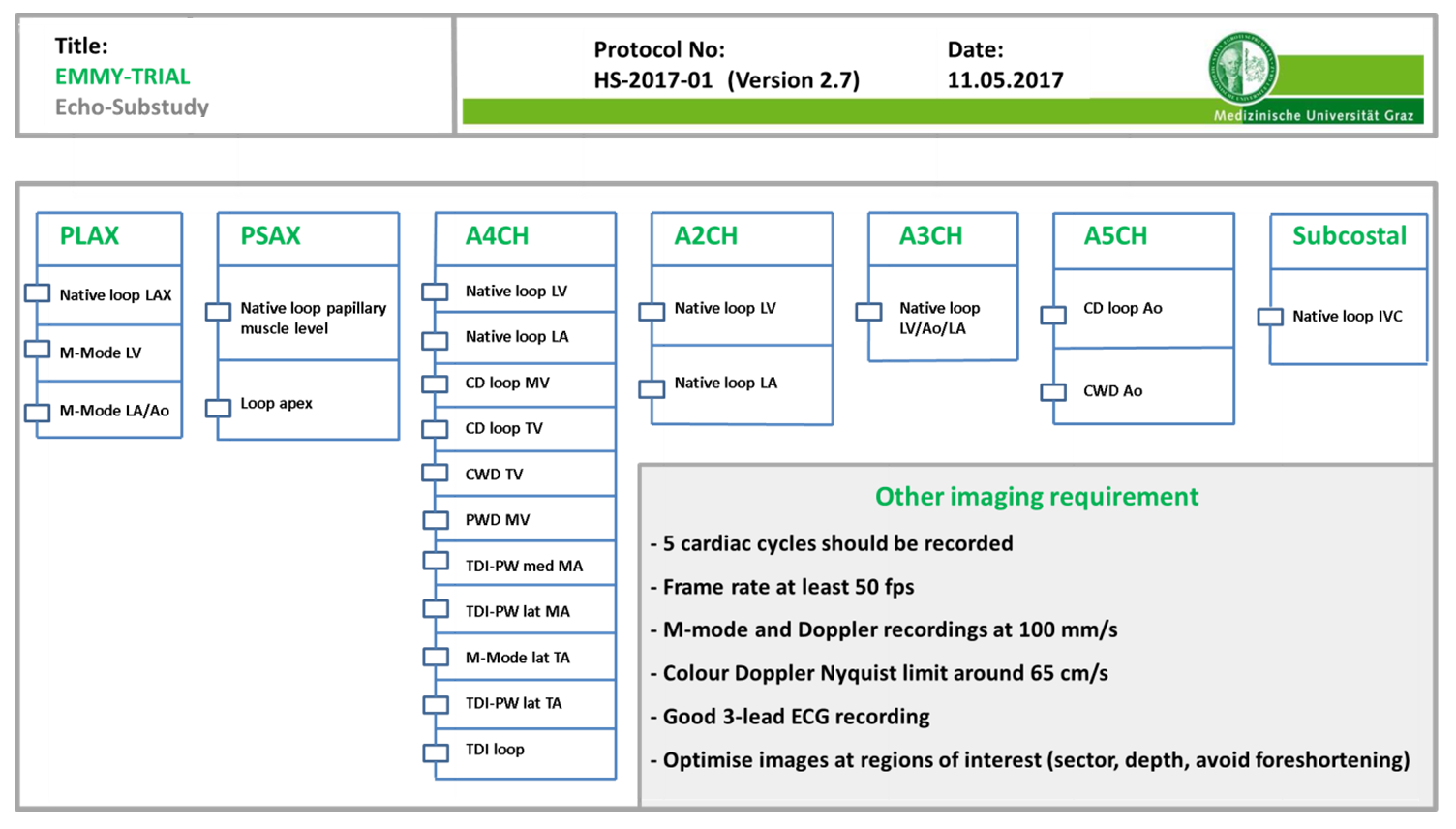
**Figure S1** Overview on the echocardiographic protocol for the EMMY post-hoc analysis

**Figure S2** Changes in echocardiographic parameters by treatment group. (A) left-ventricular ejection fraction, (B) left-ventricular global longitudinal strain, (C) left-atrial volume index, (D) E/e’, (E) tricuspid annular plane systolic excursion, (F) right-ventricular fractional area change, (G) right-ventricular freewall strain, (H) right-atrial volume index, and (I) right-atrial global longitudinal strain.


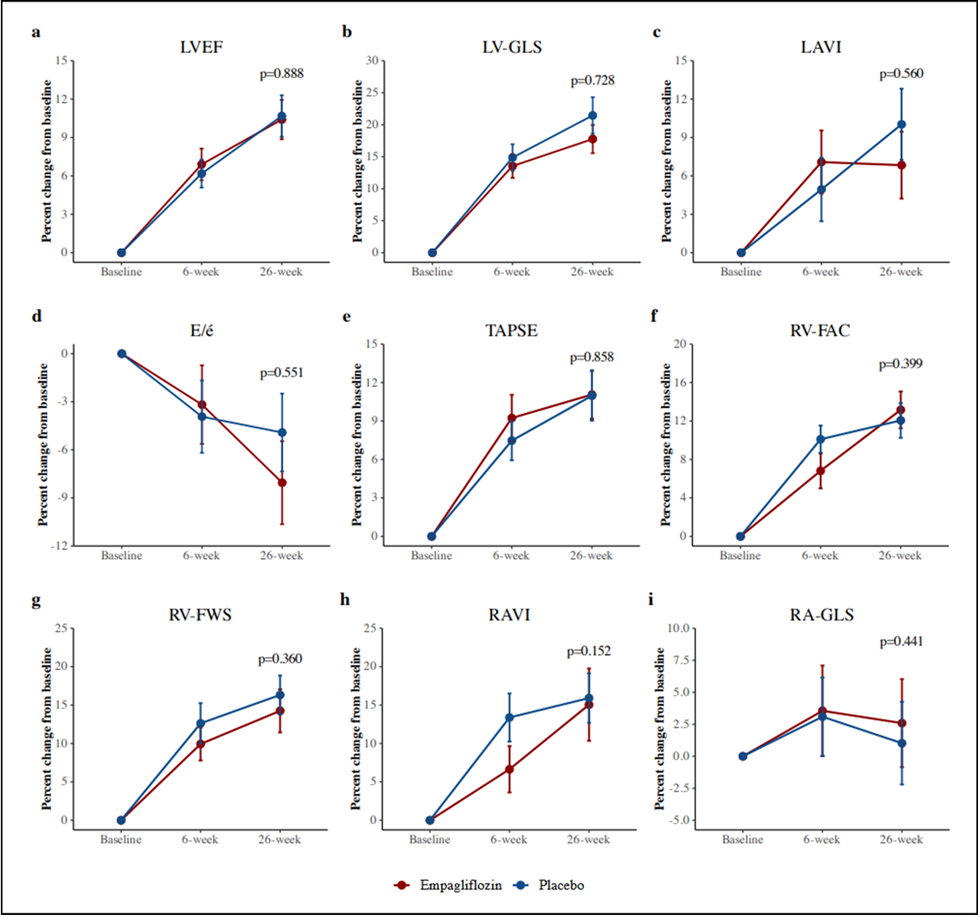

Supplement: Supplementary file 1 — Supplementary file1 (DOCX 557 KB) [file 392_2024_2523_MOESM1_ESM.docx]
